# “Ménage à trois”: the presence/absence of thyme shapes the mutualistic interaction between the host plant *Medicago truncatula* (Fabaceae) and its symbiotic bacterium *Sinorhizobium meliloti*

**DOI:** 10.1002/ece3.270

**Published:** 2012-07

**Authors:** Bodil K Ehlers, Eva Grøndahl, Joëlle Ronfort, Thomas Bataillon

**Affiliations:** 1 Biological Institute, University of Southern DenmarkCampusvej 55, 5230 Odense, Denmark; 2 INRA, UMR AGAP, Domaine de Melgueil34130 Maugio, France; 3 Bioinformatics Research Center, Aarhus University8000 Aarhus C, Denmark

**Keywords:** Fitness, genetic correlation, legume–rhizobium symbiosis

## Abstract

The long-term maintenance of specialized mutualisms remains an evolutionary puzzle. Recent focus has been on factors governing the stability of these mutualisms, including sanctions by the host, partner choice, and coevolutionary constraint, that is, the genetic correlation (*r*_G_) between fitness of both partners. So far these studies have been typically carried out in a single environment. Here, we ask if the genetic correlation between fitness of the host plant *Medicago truncatula* (Fabaceae) and its bacterial symbiont *Sinorhizobium meliloti* is affected by the presence/absence of a monoterpene (carvacrol) leached into the soil by *Thymus vulgaris*—a common plant of the Mediterranean vegetation, often co-occuring with *Medicago*. We show that the presence of carvacrol in the soil dramatically affects fitness of the rhizobial partner and increases the magnitude of *r*_G_ between plant and rhizobia fitness (*r*_G_ = 0.02 ± 0.05 vs. *r*_G_ = 0.57 ± 0.02). This finding emphasizes the importance of heterogeneity in the biotic environment for understanding the evolution of species interactions.

## Introduction

The stability of specialized mutualisms has been considered an evolutionary puzzle. As natural selection operates to maximize the fitness of each partner, a conflict of interest can undermine the maintenance of a mutualism as each partner may benefit from exploiting the other partner potentially shifting the interaction from being mutualistic to antagonistic (Sachs and Simms 2006). Mutualisms between plants and their microbial symbionts play an important role for the functioning of terrestrial communities and ecosystems. Nitrogen-fixing bacteria (rhizobia) reside in plant root organs called nodules, where they fix atmospheric nitrogen to organic nitrogen. Plants benefit from this interaction, as nitrogen is often a limiting growth factor. Bacteria in return receive photosynthetic product (sugars). The maintenance of this mutualism has received much attention and mechanisms promoting its stability include sanctioning of nonsymbiotic rhizobium by plants (Kiers et al. 2003) and partner choice, where plants form more nodules with the most beneficial rhizobium strain (Simms et al. 2006; Heath and Tiffin 2009). Genotype-by-genotype (G×G) interactions may likewise slow the breakdown of the mutualism if a genotype behaves as “cheater” when interacting with one partner genotype but as a benefactor when interacting with another partner genotype (Heath and Tiffin 2007).

Genetic correlation between traits can constrain co-evolution if the direction of the correlation is different from the vector of selection (Walsh and Blows 2009), although theory reveals that coevolutionary dynamics among interacting sets of species might be surprisingly insensitive to the direction of that correlation (Nuismer and Doebeli 2004). Heath (2010) found evidence of a trade-off, that is, a negative genetic correlation between plant and rhizobium fitness. However, genetic correlations can be affected and even reversed across different environments. For instance, legumes may bypass their interaction with nitrogen-fixing bacteria when mineral nitrogen is plentiful (Bronstein 1994). Although we expect the community context of an interaction to broadly shape its ecological and evolutionary dynamics, mutualisms are typically studied as isolated pairwise interactions and studies examining the effects of local environment on plant–soil symbionts interactions remain rare (but see Piculell et al. 2008; Heath et al. 2010; Heath and Lau 2011).

The barrel medic (*Medicago truncatula*) is a selfing annual growing naturally in the Mediterranean garrigue vegetation. Aromatic plants, especially species from the plant family *Lamiaceae*, often dominate this type of vegetation and produce monoterpenes as a main component of their essential oil that enter the local soil by leaching from leaf litter. These monoterpenes can affect the amount and availability of nitrogen in the soil via their effect on heterotrophic soil microorganisms, by increasing nitrogen immobilization and reducing nitrogen mineralization (White 1994; Pavolainen et al. 1998). The effect of monoterpenes on these processes varies with their concentration but the inhibition of the nitrification process occurs with monoterpene concentrations as low as 10–125 µg g^−1^: well within the range of what can be found in natural conditions (White 1994).

One aromatic plant commonly dominating the garrigue vegetation is wild thyme (*Thymus vulgaris*). Vegetation analysis from 10 different thyme dominated plant communities showed that wild thyme coexisted with 43 different legume species, including five species of the genus *Medicago* among them is *M. truncatula* (B. K. Ehlers, unpubl. data). *Medicago truncatula* also naturally occurs along vineyards or in abandoned fields where thyme is not present. Here, we use this ecological setting and test if presence/absence of a thyme monoterpene (carvacrol) in the soil can affect the mutualistic interaction between *M. truncatula* and its nitrogen-fixing root symbiont *Sinorhizobium meliloti*.

## Material and Methods

### Experimental design

We grew six different genotypes of *M. truncatula* in association with either no rhizobia (control) or one of two different rhizobia mixes. All six *M. truncatula* genotypes were highly inbred genotypes collected from populations where *M. truncatula* is naturally occurring. As most *M. truncatula* are naturally infected by several different strains (Bailly et al. 2006), we used two mixes of rhizobium comprising each two different strains of *S. meliloti*. Each *Medicago*×*rhizobia* mix (hereafter *M*×*R*) combination was grown in two soil treatments (soil treated with carvacrol or without) in a fully factorial design in the greenhouse (with five replicates per *M*×*R* combination for a total of 180 plants).

### *Medicago* and *Rhizobium* genotypes origin

Three nonrelated *M. truncatula* genotypes (NA1021, NA216, NA632) originated from one site in Southern France (La Nautique), where *M. truncatula* and Thyme (*T. vulgaris*) are naturally occurring, have been coexisting for years and are commonly observed growing with an interplant distance of just 10–20 cm (B. K. Ehlers, pers. obs.). The remaining genotypes (A17, DZA012, F20089-B) originated each from a different population. Sterile *M. truncatula* seeds from all genotypes inbred lines obtained by at least two generations of selfing of highly homozygous genotypes collected in nature. Detailed information about the genotypes is publicly available at http://www1.montpellier.inra.fr/BRC-MTR. Preliminary SNP genotyping information shows that all inbred lines are genetically nonrelated and that the mean pairwise number of differences is homogeneous across pairs of genotypes between/within locations (J. Ronfort, unpubl. data). All inbred lines have been grown in a common greenhouse environment for at least two generations so the scope for maternal effects “imported” from different field conditions and inflating the variance attributed to genotype effects is minimal.

One rhizobia mix contained two *S. meliloti* genotypes previously isolated from soil from the site “La Nautique,” where three *Medicago* genotypes were also sampled. The other mix was composed of two *S. meliloti* genotypes originating from two different sites in Southern France, where thyme is known not to be present. All rhizobium genotypes were kindly provided by Dr. Katy Heath (University of Illinois) and the isolation of the strain from soil is explained in detail in Heath (2010). Forty-eight hours before inoculation, all rhizobium genotypes were grown separately in sterile liquid cultures (Heath 2010). A similar density of each rhizobium strain (10^6^ cells/mL, as measured by optical density count) was assured before mixing. Each plant received 1 mL of inoculum, and each control plant received 1 mL of liquid growth media without rhizobium.

### Soil preparation

Standard greenhouse soil (Pindstrup mix no. 2) was mixed with one-third sand, steam sterilized and kept in sealed plastic bags until use. Soil containing the thyme monoterpene “carvacrol” was prepared by adding 35 µl of liquid pure carvacrol (Sigma-Aldrich Denmark A/S, Broendby, Denmark) per 100 g soil (dry weight). This concentration of carvacrol (325 µg g^−1^ soil) is within the range found under natural field conditions (White 1991; Grøndahl and Ehlers 2008). Carvacrol can make up to 80% of the essential oil of naturally occurring thyme plants, and is the dominating mono-terpene produced in the geographic region where the site “La Nautique” is located (Stahl-Biskup 2002; Thompson 2005a).

Liquid carvacrol was mixed in petri dishes with filter paper (Filtrak paper sheet, 17.95 g m^2^) cut in pieces of approximately 1 cm^2^ and sealed with plastic film for 24 h after which all liquid had soaked into the filter papers. Filter paper was then mixed thoroughly into soil using one single container of soil. Soil with filter paper was sealed with plastic and left for another 24 h to homogenize the concentration of carvacrol before adding soil to individual germination trays and pots.

### Germination and potting

Seeds were scarified and kept cold (5°C) for two weeks and then added to germination trays containing soil with and without carvacrol. Trays were covered with plastic and placed in a cold greenhouse (8°C). After 10 days, when seedlings began to emerge, plastic cover was removed and seedlings were moved to a warm greenhouse (28°C) with day length of 18 h. One week later, seedlings were transplanted to individual pots. Pots were inoculated with rhizobium eight days later.

Pots were placed on tables in the greenhouse—each table containing separate sub blocks that were isolated from each other by a distance of 0.5 m and each sub block was raised from the table to assure that run off water after watering could not enter adjacent pots from below. This was done to minimize the risk of contamination of rhizobium to pot with different rhizobium mix and pots without rhizobium. No fertilizer was added to the soil mix, and plants were watered daily with a hose. When the first fruits began to develop watering was reduced to every other day. This was to mimic a field situation where drought period typically begin around the time of fruiting. Two months after inoculation, all plants were harvested. Number of fruits and number of nodules were counted on each individual plant and used to estimate, respectively, plant and rhizobial fitness. Plants grown without rhizobium had their roots carefully examined for presence of nodules to assess if contamination had occurred during the course of the experiment.

### Statistical analysis

Univariate analyses of variance (ANOVAs) were used to estimate genetic and environmental component of plant fitness (measured through fruit set) and rhizobial fitness (nodule number); and to test for the effect of *Medicago* genotype, soil treatment, rhizobium, and their interaction.

Broad-sense genetic correlations between fruit set and nodule number among all 12 *Medicago* genotype ×*Rhizobium* mix associations were estimated within each soil treatment. We note that defining these genetic correlations using the set of *Medicago* genotype ×*Rhizobium* mix associations stretches the definition of a genetic correlation, which typically is defined as the correlation between genotypic values for two traits over a set of genotypes. However, in the context of a pairwise symbiosis involving a single plant genotype and possibly a mix of genetically heterogeneous rhizobia, we feel it allows asking whether the fitness component of each entity is genotype (or plant genotype × bacterial genotype mix) dependent. It also makes sense as it is still defined within one environment over a set of defined repeatable interacting entities (*Medicago* genotype ×*Rhizobium* mix associations, hereafter *M*×*R* associations).

The experimental design we used was perfectly balanced, and both rhizobia and plant genotypes we used are clones/mix of clones; therefore, estimates of environmental and genetic variance/covariance matrices can be obtained directly from a multivariate analysis of variance (MANOVA) with fruit set and nodule numbers as joint response variables and *M*×*R* associations treated as a single random factor (Lynch and Walsh 1998). More specifically, under this design, just as in the univariate case, method-of-moment estimates for the (causal) broad-sense genetic (**Cov**_**G**_) and environmental (**Cov**_**E**_) variance–covariance matrices can be obtained as a function of the (observable) main effect (**H**) and residual (**E**) matrices of cross products from the MANOVA table (we use **bold** type to denote matrices).

In our design, *g* = 12 *M*×*R* genotypic associations and *k* = 5 replicates per association are used within each environment, yielding the method-of-moment estimates for **Cov**_**E**_ and **Cov**_**G**_:


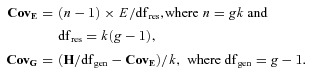


From these, we obtained the broad-sense environmental (*r*_E_) and genetic correlations as (*r*_G_) as a function of **Cov**_**G**_ and **Cov**_**E**_. Approximate SEs for the broad-sense genetic and environmental correlation were obtained by jackknifing over the 12 *M*×*R* associations.

## Results

We found that both *Medicago* genotypes and Rhizobium strain mix had a highly significant main effect on the fitness components of both partners (Table 1). Thus, the level of replication in our experiment was adequate to reliably detect the fitness differences occurring among genotypic *M*×*R* associations.

**Table 1:** Summary of three-way ANOVA on number of nodules and ln (number of fruit)

		Number of nodules(*R*^2^ = 0.38)		ln (fruit set)(*R*^2^ = 0.41)	
			
Source	df	Sum of squares	*F* (*P*-value)a)	Sum of squares	*F* (*P*-value)
*Medicago* genotype (M)	5	32,559	**6.57 (<10^−4^)**	10.63	**11.3 (<10^−7^)**
*Rhizobium* mix (R)	1	4246	**4.29 (0.04)**	1.66	**1.66 (0.004)**
Soil treatment (ST)	1	407	0.41 (0.52)	0.0001	0.004 (0.98)
M × ST	5	12,048	**2.43 (0.04)**	0.204	0.216 (0.95)
M × R	5	5970	1.21 (0.31)	0.442	0.469 (0.80)
R × ST	1	2189	2.20 (0.14)	0.0002	0.001 (0.98)
M × R × ST	5	3399	0.68 (0.64)	0.174	0.184 (0.96)
Error	99	98,077		18.65	

a) Factors that are significant at the 0.05 level are in bold.

**Figure 1:**
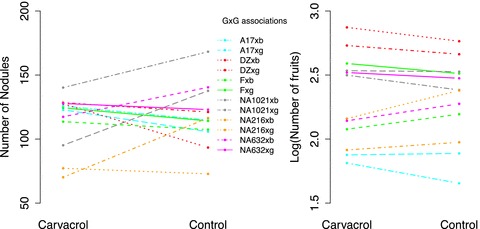
Reaction norms of fitness components in soil treated with thyme terpene (carvacrol) and control soil (without thyme terpene). All reaction norms are reported as the mean of *n* = 5 independent replicates for each 12 G×G (*Medicago* by *Rhizobium* mix) association. *Medicago* genotypes are coded by their geographic origin (see Material and Methods for full name of genotypes). *Rhizobium* mix is coded as either “g” (naïve to thyme) or “b” (collected in soil where Thyme was present). Standard errors around the mean (SEM) are omitted from the graph to increase readability but SEM were very homogeneous among G×G associations: SEM = 19–20 for number of nodules on either control or carvacrol soil. SEM = 0.3 (control) and SEM = 0.2 (carvacrol) for ln (number of fruits).

For *Rhizobium* fitness, a significant interaction between plant genotype and soil treatment was caused by the fact that *M. truncatula* genotypes originating from La Nautique (genotypes NA1021, NA216 and NA632 coexisting with thyme; Fig. 1) produced fewer nodules on “thyme soil” and more on “no-thyme” soil (*t*_1_ = 2.35, *P* = 0.02), whereas the reverse was true for the three remaining *Medicago* genotypes (*t*_1_ = 2.13, *P* = 0.03). We did not detect a significant interaction between *Medicago* genotype and *Rhizobium* mix. However, a post hoc comparison showed that *Medicago* genotypes from “La Nautique” had more nodules when grown with their “home”*Rhizobia* mix than with the other *Rhizobium* (*t*_1_ = 2.46, *P* = 0.01; Fig. 1), whereas the remaining three *Medicago* had similar amount of nodules irrespective of *Rhizobium* mix (*t*_1_ = 0.47, *P* = 0.63; Fig. 1).

Fruit set was strongly plant genotype dependent. Fruit set was reduced in plants without *Rhizobium*, and more so on soil containing carvacrol (Table 1). The main effect of soil treatment was only significant when including the control (uninoculated) plants in the analysis.

**Figure 2:**
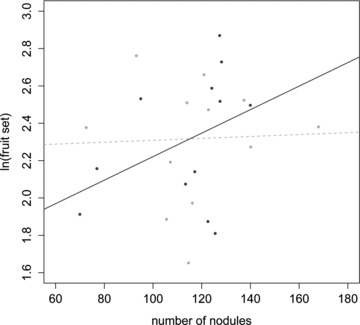
Gray circles depict the joint estimates of genotypic values for each *M*×*R* association and the major axis regression (gray dotted) line in the “no carvacrol” soil environment. Black circles depict the joint estimates of genotypic values and the major axis (black) regression line in the “carvacrol” soil environment.

The genetic correlation between *Medicago* genotype and *Rhizobium* strains components of fitness was strongly affected by soil treatment. A positive correlation between rhizobium and plant fitness was found on carvacrol soil, whereas this correlation was virtually zero on control soil (Table 2; Fig. 2).

**Table 2:** Genetic and environmental correlations between fitness components of both partners

Environment	*n*	*r*_G_ (± SE)	*r*_E_ (± SE)
Control soil (no carvacrol)	12	0.02 (± 0.05)	0.08 (± 0.1)
Carvacrol soil	12	0.57 (± 0.02)	0.07 (± 0.08)

*n*, number of *M*×*R* genotypes associations used to estimate broad-sense genetic correlation (*r*_G_) and environmental correlation (*r*_E_) in each environment.

## Discussion

Recent studies emphasizethe importance of the community context for understanding species interactions (Thompson 2005b; Agrawal et al. 2007). This context is crucial when considering coevolved mutualistic interactions where the fitness of individual partners varies across different environments thereby potentially affecting the stability of the interaction. So far, empirical studies of the effect of the local environment on the fitness of both partners in a mutualistic interaction remain scarce (Piculell et al. 2008; Heath et al. 2010). Here, we show that rhizobium fitness components and the broad-sense genetic correlation between *Medicago* plant fitness and their nitrogen-fixing root symbiont fitness is strongly affected by the presence of a thyme monoterpene (carvacrol) in the soil.

Given that the presence of multiple rhizobia within a single *Medicago* genotype is very common in nature (Bailly et al. 2006), we chose to use mixes comprising two rhizobia strains instead of pure cultures to inoculate *M. truncatula*. Given that we use repeatable mixes of genotypes, we argue that the concept of broad-sense genetic and environmental correlations (*r*_G_ and *r*_E_ estimates; Table 2) is still valid in this situation (but see also below).

One could also argue that *M. truncatula* genotypes are effectively infected by only one strain of the rhizobial mix when grown on soil with/without carvacrol and that differences in *r*_G_ reported here merely reflect the fact that *r*_G_s are defined over a slightly different set of *M*×*R* associations in each soil environment. We currently do not have a detailed molecular genotyping system for the rhizobia strains, and thus cannot formerly rule out this hypothesis. However, we find it unlikely given that we specifically chose a set of rhizobia strains that were proven to be all nodulating and beneficial as single culture (Heath 2010).

*Medicago truncatula*, *T. vulgaris*, and *S. meliloti* commonly co-occur in nature and, barren the cautionary remarks above, our results suggests that the patchy environment created by presence/absence of thyme among sites where partners interact will affect the evolutionary dynamic of the *M. truncatula*–*S. meliloti* mutualism. The presence of a thyme monoterpene in the soil drives a positive correlation between plant and rhizobia fitness. This is interesting for two reasons. First, there is a general belief that such correlations should be negative (Sachs and Simms 2006; Heath 2010). Second, the existence of an environment where the correlation between partner fitness is positive can potentially stabilize this mutualism by aligning the fitness interest of the two partners.

We did not test if the fitness of free-living *S. meliloti* strains was affected by carvacrol in the soil. However, essential oils from aromatic plants are renowned for their antimicrobial activity and the inhibitory effect of essential oil—including carvacrol—on the growth of free-living *Rhizobium leguminosarum* is documented (Sivropoulou et al. 1995, 1996). *Rhizobium* may benefit from the interaction with plants as the nodules create an environment sheltered from the mono-terpenes.

In summary, our results suggest that the fitness performances of partners of a tight mutualism (involving here the model legume *M. truncatula* and associated specific rhizobia) can be affected by the presence/absence of a third biotic species (thyme). One caveat remains: the genetic correlations and reactions norms we estimated are defined over the whole set of G×G associations used as a virtual reference population. Depending on the spatial level considered and the level of genotypic diversity available within population for each partner, only a fraction of these G×G associations may actually “meet and compete” in nature. Future studies employing a similar G×G×E factorial design but encompassing more genotypes per geographic location will have the potential to quantify the relative importance of heterogeneity in the biotic and abiotic environment for shaping local adaptation and coevolution of interacting species (Nuismer and Gandon 2008).
